# NMR Studies on Li^+^, Na^+^ and K^+^ Complexes of Orthoester Cryptand *o*-Me_2_-1.1.1

**DOI:** 10.3390/ijms160920641

**Published:** 2015-08-31

**Authors:** René-Chris Brachvogel, Harald Maid, Max von Delius

**Affiliations:** Department of Chemistry and Pharmacy, Friedrich-Alexander University Erlangen-Nürnberg (FAU), Henkestr. 42, 91054 Erlangen, Germany; E-Mails: rene.brachvogel@fau.de (R.-C.B.); harald.maid@fau.de (H.M.)

**Keywords:** supramolecular chemistry, cryptands, orthoesters, dynamic covalent chemistry, NMR spectroscopy, host-guest complexes

## Abstract

Cryptands, a class of three-dimensional macrobicyclic hosts ideally suited for accommodating small guest ions, have played an important role in the early development of supramolecular chemistry. In contrast to related two-dimensional crown ethers, cryptands have so far only found limited applications, owing in large part to their relatively inefficient multistep synthesis. We have recently described a convenient one-pot, template synthesis of cryptands based on *O*,*O*,*O*-orthoesters acting as bridgeheads. Here we report variable-temperature, ^1^H-1D EXSY and titration NMR studies on lithium, sodium, and potassium complexes of one such cryptand (***o*-Me_2_-1.1.1**). Our results indicate that lithium and sodium ions fit into the central cavity of the cryptand, resulting in a comparably high binding affinity and slow exchange with the bulk. The potassium ion binds instead in an *exo* fashion, resulting in relatively weak binding, associated with fast exchange kinetics. Collectively, these results indicate that orthoester cryptands such as ***o*-Me_2_-1.1.1** exhibit thermodynamic and kinetic properties in between those typically found for classical crown ethers and cryptands and that future efforts should be directed towards increasing the binding constants.

## 1. Introduction

Crown ethers (macrocyclic oligomers of ethylene glycol) [1,2] and cryptands (bicyclic structures made up of oligoethylene glycol arms and trialkylamine bridgeheads) [3,4,5,6] are iconic supramolecular hosts that through their interaction with small guest ions have contributed significantly to our current understanding of non-covalent interactions. The archetypal guests complexed by these hosts are alkali metal ions and the kinetics, as well as the thermodynamics of this association process, have been comprehensively studied and reviewed [7]. Prior to 2015, only few studies reported significant variations to the crucial bridgehead architecture of cryptands. For example, Coxon and Stoddart have described the multi-step synthesis (total yield less than 1%) of a 1.1.1-tris(hydroxymethyl)ethane-capped cryptand that exclusively possesses oxygen donor atoms [8]. Parsons and coworkers have synthesized related, yet less symmetric, macrobicycles in which two glycerol motifs act as bridgeheads [9,10,11]. Saalfrank and coworkers have self-assembled metallocryptates in which iron ions act as bridgeheads and a mix of nitrogen and oxygen donors is available for cation binding [12,13]. Lehn and Nelson have prepared tripodal imine-based bimetallic cryptates, featuring mainly nitrogen donors [14,15,16,17] and Voloshin, as well as others have reported studies on kinetically-inert “clathrochelates”, in which three glyoxime-type ligands are capped by borate bridgeheads [18,19,20,21].

**Figure 1:**
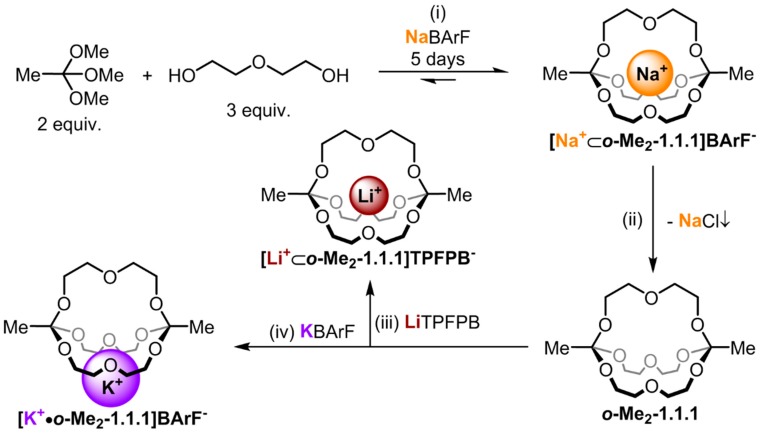
Synthesis route for the different alkali metal complexes. (**i**) 5% TFA, MS 4 Å, CDCl_3_ (10 mM in respect to NaBArF (sodium tetrakis[3,5-bis(trifluoromethyl)phenyl]borate)), RT; (**ii**) Lewatit^®^ MP-64 anion exchange resin, CDCl_3_, 6h, RT; (**iii**) 1.0 equiv. LiTPFPB (lithium tetrakis(pentafluorophenyl) borate ethyl etherate), CDCl_3_, RT; (**iv**) 1.0 equiv. KBArF (potassium tetrakis[3,5-bis(trifluoromethyl)phenyl]borate), CDCl_3_, RT.

We have recently described a one-pot, template synthesis of monometallic cryptates based on *O*,*O*,*O*-orthoester bridgeheads [22]. As shown in Figure 1, both the unique structural (tripodal geometry) [23] and dynamic (acid-catalyzed exchange with alcohols) [24] features of orthoesters are responsible for the remarkable efficiency of this self-assembly process. Due to the fact that this new class of cryptands is constitutionally dynamic in the presence of acid and the cage structure can be disintegrated at low pH, we anticipate that rather unique curiosity-driven (subcomponent self-sorting and systems chemistry), as well as application-oriented (controlled guest release and drug delivery), studies can be pursued with this new class of compounds. However, to engage in such endeavors, a detailed knowledge of the properties of these compounds and their accommodation of guest ions is needed. In our initial communication on the sodium-templated self-assembly of orthoester cryptates [22], we did only report preliminary data on the thermodynamics and kinetics of the binding of different metal ions with orthoester cryptand ***o*-Me_2_-1.1.1**. Herein, we report more comprehensive physicochemical data and we discuss the implications of our findings on future research directions.

## 2. Results and Discussion

Four compounds had to be prepared to carry out the NMR studies described herein: ***o*-Me_2_-1.1.1**, **[Na^+^**⊂***o*-Me_2_-1.1.1]BArF^−^**, **[Li^+^**⊂***o*-Me_2_-1.1.1]TPFPB^−^** and **[K^+^•*o*-Me_2_-1.1.1]BArF^−^**. As shown in Figure 1, cryptate **[Na^+^**⊂***o*-Me_2_-1.1.1]BArF^−^** was prepared in 67% yield using the templated self-assembly reaction (reaction scale typically 50 mg in respect to isolated product). Cryptand ***o*-Me_2_-1.1.1** was obtained by treating a chloroform solution of **[Na^+^**⊂***o*-Me_2_-1.1.1]BArF^−^** with anion exchange resin Lewatite^®^ MP-64, which led to the precipitation of NaCl, along with a solution of the desired empty cage (reaction scale typically 10 mg in respect to product; due to its high sensitivity to acid-catalyzed hydrolysis, cryptand ***o*-Me_2_-1.1.1** is best freshly prepared). The lithium and potassium complexes **[Li^+^**⊂***o*-Me_2_-1.1.1]TPFPB^−^** and **[K^+^•*o*-Me_2_-1.1.1]BArF^−^**, respectively, were obtained by titration of the corresponding metal salts to cryptand ***o*-Me_2_-1.1.1**. For details regarding these syntheses, please refer to the experimental section.

**Figure 2:**
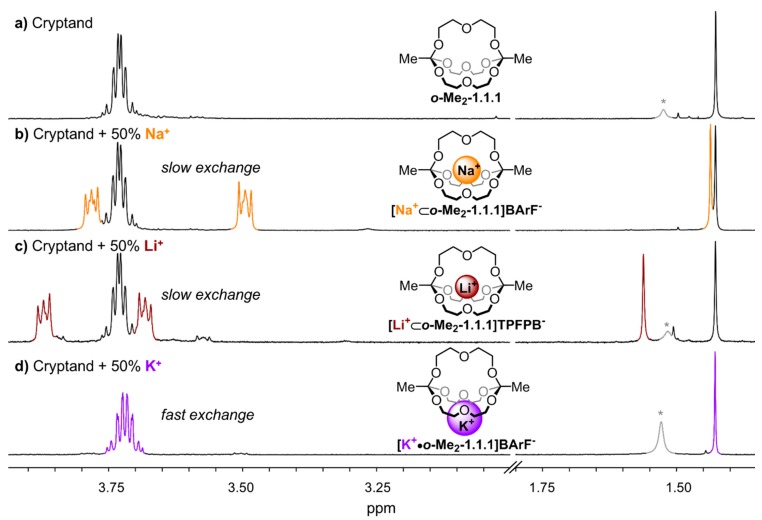
Partial ^1^H NMR (400 MHz, 298 K, CDCl_3_) stack plot; (**a**) only ***o*-Me_2_-1.1.1**; (**b**) 1:1 mixture of ***o*-Me_2_-1.1.1** and **[Na^+^**⊂***o*-Me_2_-1.1.1]BArF^−^**; (**c**) 1:1 mixture of ***o*-Me_2_-1.1.1** and **[Li^+^**⊂***o*-Me_2_-1.1.1]TPFPB^−^**; and (**d**) 1:1 mixture of ***o*-Me_2_-1.1.1** and **[K^+^•*o*-Me_2_-1.1.1]BArF^−^**. *: Peaks in grey color correspond to water.

Figure 2 gives an overview of the ^1^H NMR spectra of the different investigated alkali metal complexes in chloroform. The “empty” cryptand ***o*-Me_2_-1.1.1** exhibits a singlet at 1.43 ppm and a multiplet at 3.73 ppm (Figure 2a). Fundamental differences were observed in the spectra that were obtained when 0.5 equivalents of different metal salts (NaBArF, LiTPFPB, KBArF) were added to ***o*-Me_2_-1.1.1** via titration. For the sodium and lithium complexes (Figure 2b,c), we observed NMR spectra indicative for slow cation exchange (different sets of signals for the cryptates and for the cryptand), whereas fast exchange was observed for the potassium complex (one average signal set, see Figure 2d). These results indicate at a qualitative level that the kinetics for the process of one metal ion hopping from one orthoester cage into another are significantly slower for lithium and sodium ions than for potassium ions. To investigate whether this seemingly drastic difference in the kinetics of cation exchange is also reflected in the thermodynamic binding strength, we performed ^1^H NMR titrations in solvent acetonitrile. This solvent was chosen because all studied metal salts were soluble therein and because it facilitated fast cation exchange on the NMR timescale, so that binding isotherms could be obtained. Using 1D EXSY [25] (exchange spectroscopy) and VT (variable temperature) NMR spectroscopy we also studied the kinetics of cation exchange at a quantitative level. These measurements are described for each alkali metal individually in Section 2.1, Section 2.2 and Section 2.3, while Section 2.4 discusses the effect of different counter-anions, and Section 2.5 will provide an overview and a discussion of the results.

### 2.1. Thermodynamic and Kinetic Properties of **[Na^+^**⊂**o-Me_2_-1.1.1]BArF^−^**

In previously-published NMR titration experiments [22], we treated empty cryptand ***o*-Me_2_-1.1.1** with NaBArF in solvent CDCl_3_ (saturated with D_2_O). Under these conditions, we observed slow exchange on the NMR time scale and we could show by competition experiments with classic cryptand [2.2.1] and crown ether 15-crown-5 that the binding constant for this orthoester cryptand lies in between the binding constants for the two competing classic macro(bi)cyclic hosts.

**Figure 3:**
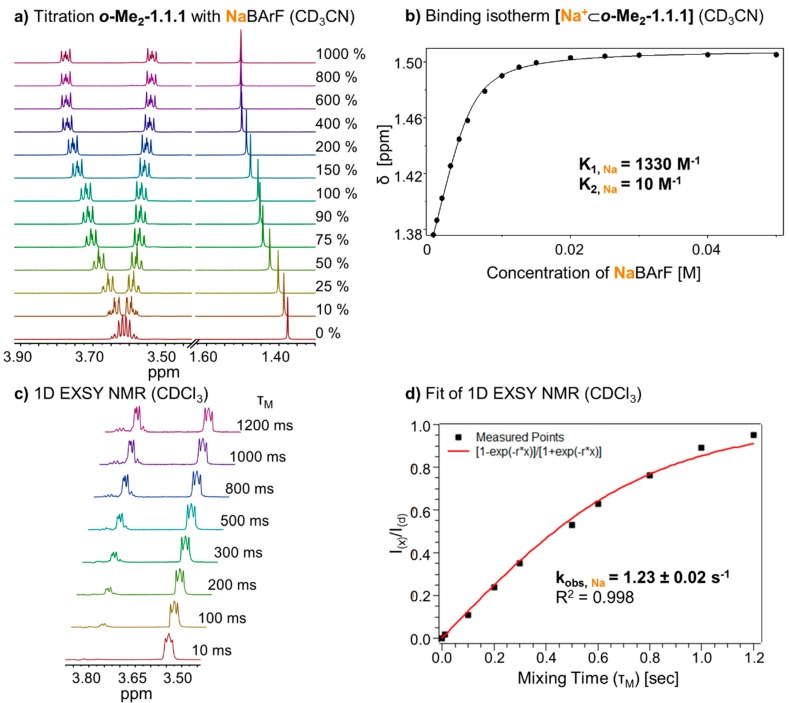
(**a**) Partial ^1^H NMR (400 MHz, 298 K, CD_3_CN) stack plot of ***o*-Me_2_-1.1.1** (5 mM) titration with NaBArF from 0 to 1000 mol %; (**b**) Fit of binding isotherm of **[Na^+^**⊂***o*-Me_2_-1.1.1]** (K_1_ for 1:1 complex, K_2_ for 1:2 complex, estimated error: <10%); (**c**) ^1^H-1D EXSY experiment (400 MHz, 298 K, CDCl_3_) with different mixing times (τ_m_); and (**d**) Fit of ^1^H-1D EXSY experiment and determination of exchange rate for **[Na^+^**⊂***o*-Me_2_-1.1.1]BArF^−^**.

To obtain more meaningful thermodynamic information on metal binding, we proceeded to titrate a solution of NaBArF (0 to 1000 mol %) to ***o*-Me_2_-1.1.1** in solvent CD_3_CN (Figure 3a). In this case, fast exchange on the NMR time scale allowed a quantitative analysis of the system’s thermodynamics. By fitting the data of the binding isotherm, using program HypNMR [25], we were able to determine the binding constant of the complex. Interestingly, an excellent fit of the data could only be obtained when both a 1:1 and a 1:2 complex was taken into account (“1:2” indicating one metal and two cryptands). As shown in Figure 3b, the resulting association constants (K_A, **Na**_) in CD_3_CN are 1330 M^−1^ (K_1_) for the formation of the 1:1 complex and 10 M^−1^ (K_2_) for the equilibrium between the 1:1 and the 1:2 complex [26]. It has to be emphasized that a consideration of the 1:2 complex was only necessary to achieve a truly excellent fit of the data and that K_2_ is (relatively) so small that the 1:2 complex is only present in noticeable quantities during the “early” stage of the titration. For a plot that shows the composition of the system during the titration, please refer to the Supplementary Figure S4.

To investigate the kinetic behavior of the sodium complex and determine the rate of cation exchange, we prepared a 1:1 mixture of **[Na^+^**⊂***o*-Me_2_-1.1.1]BarF^−^** and ***o*-Me_2_-1.1.1** in CDCl_3_. ^1^H 1D EXSY NMR spectra were recorded from this sample, using different mixing times (τ_m_) from 10 ms to 1200 ms in CDCl_3_ (Figure 3c). Due to selective excitation of the peak at 3.5 ppm, the correlated peak at 3.8 ppm increased with longer mixing times. By plotting the quotient of the integrals from the EXSY peak (I_(X)_) and excited peak (I_(d)_), and fitting this curve (see Figure 3d and Section 3.2.3 for the underlying equation), we obtained an exchange rate (k_obs, Na_) [27] of 1.23 ± 0.02 s^−1^ [28,29,30]. This exchange rate k_obs_ is a measure for how frequently a given sodium cation changes position from one cryptand host to another. In other words, for cryptate **[Na^+^**⊂***o*-Me_2_-1.1.1]BarF^−^** such a “hopping” event occurs on average one time per second (under the given experimental conditions), which is rather slow for such a process.

We also recorded variable temperature NMR (VT NMR) spectra of cryptate **[Na^+^**⊂***o*-Me_2_-1.1.1]BarF^−^** in CD_2_Cl_2_ from 25 °C down to −75 °C (see Supplementary Figure S1). Interestingly, we observed coalescence of the CH_2_ group closer to the bridgehead at −55 °C (corresponding to a ΔG^‡^ of 41.7 kJ/mol) [31], whereas the signal of the CH_2_ group in the α position to the central ether oxygen did only broaden, but not coalesce, when cooling further to −75 °C.

### 2.2. Thermodynamic and Kinetic Properties of **[Li^+^**⊂**o-Me_2_-1.1.1]TPFPB^−^**

**Figure 4:**
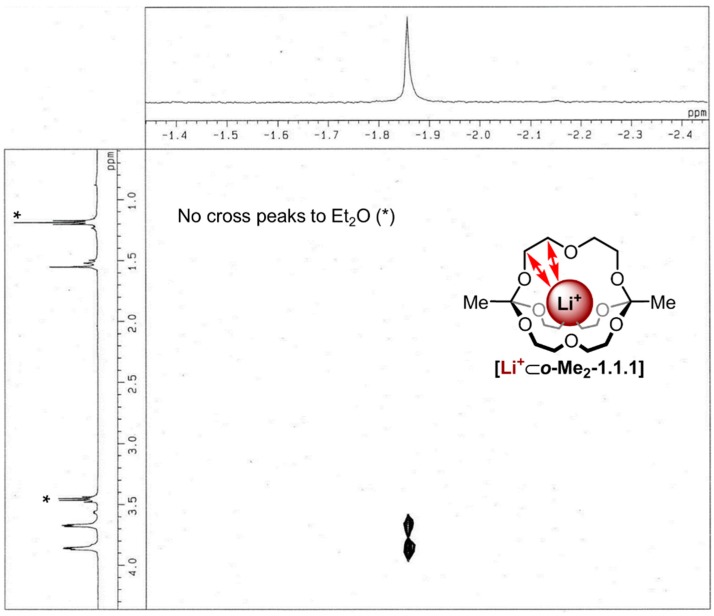
^1^H/^7^Li-HOESY NMR spectrum (500 MHz, 298 K, CDCl_3_, τ_m_: 800 ms) of **[Li^+^**⊂***o*-Me_2_-1.1.1]TPFPB^−^**, *: Peaks correspond to Et_2_O.

Cryptate **[Li^+^**⊂***o*-Me_2_-1.1.1]TPFPB^−^** was obtained by addition of the lithium salt LiTPFPB (lithium tetrakispentafluorophenyl borate diethyl etherate) to ***o*-Me_2_-1.1.1**. The ^1^H NMR spectrum of this cryptate showed a singlet at 1.56 ppm and two triplets of higher order at 3.87 and 3.68 ppm in CDCl_3_ (see Figure 2c). Titration of LiTPFPB to ***o*-Me_2_-1.1.1** in CDCl_3_ gave rise to two sets of peaks for cryptate and cryptand, respectively, indicating a slow exchange on the NMR time scale. As we were not able to obtain suitable single crystals of this compound, we aimed to provide evidence for the *endo* binding of the metal ion in this complex by ^1^H/^7^Li-HOESY NMR spectroscopy [32]. As shown in Figure 4, pronounced ^1^H/^7^Li cross-peaks were observed for both CH_2_ groups of the diethylene glycol chain, thus indicating close spatial proximity between the lithium ion and these groups. The fact that residual diethyl ether showed no cross peaks to the lithium signal indicates the lithium ion is indeed buried inside the cavity of the cage and is not rapidly “shuttling” around the cage in an *exo* fashion.

**Figure 5:**
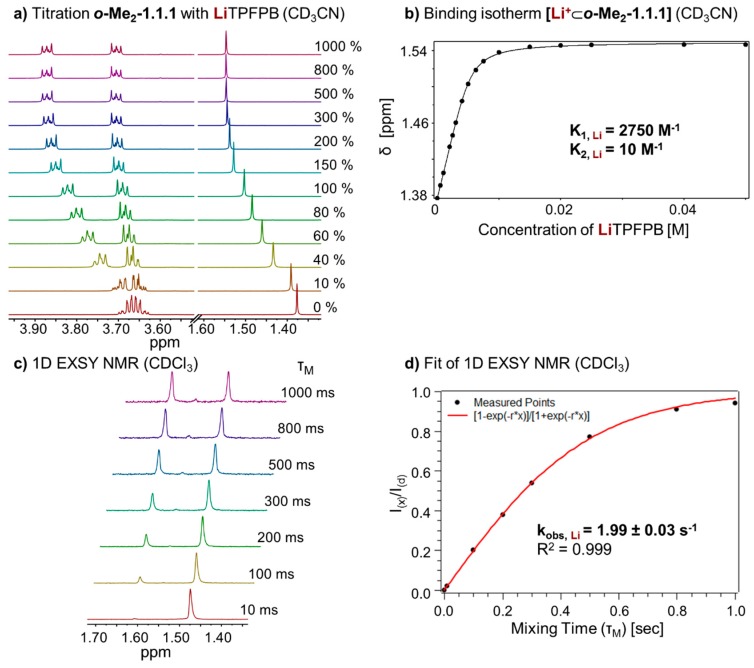
(**a**) Partial ^1^H NMR (400 MHz, 298 K, CD_3_CN) stack plot of ***o*-Me_2_-1.1.1** (5 mM) titration with LiTPFPB from 0 to 1000 mol %; (**b**) Binding isotherm of **[Li^+^**⊂***o*-Me_2_-1.1.1]** (K_1_ for 1:1 complex, K_2_ for 1:2, complex estimated error: <10%); (**c**) ^1^H-1D EXSY experiment (400 MHz, 298 K, CDCl_3_) with different mixing times (τ_m_); and (**d**) Fit of ^1^H-1D EXSY experiment and determination of exchange rate for **[Li^+^**⊂***o*-Me_2_-1.1.1]TPFPB^−^**.

To investigate the thermodynamic properties of the **[Li^+^**⊂***o*-Me_2_-1.1.1]TPFPB^−^** complex, we performed a titration of LiTPFPB to ***o*-Me_2_-1.1.1** in CD_3_CN (Figure 5a). Due to fast exchange in this solvent, it was possible to determine the binding constant (K_A, **Li**_) of the complex by fitting the binding isotherm (Figure 5b). Again, a 1:1 and 1:2 (one guest and 2 hosts) model was taken into account using program HypNMR [25]. The resulting binding constants (K_A, **Li**_) for the two complexes in CD_3_CN are 2750 M^−1^ for the 1:1 complex and 10 M^−1^ for the 1:2 complex (see Supplementary Figure S5).

In analogy to the sodium complex, we studied the kinetics of cation exchange between complex **[Li^+^**⊂***o*-Me_2_-1.1.1]TPFPB^−^** and cryptand ***o*-Me_2_-1.1.1**. Recording ^1^H-1D EXSY NMR spectra (Figure 5c) and fitting of the data (Figure 5d) allowed us to determine an exchange rate (k_obs, **Li**_) of 1.99 ± 0.03 s^−1^ for this complex.

We also recorded VT-NMR of complex **[Li^+^**⊂***o*-Me_2_-1.1.1]TPFPB^−^** in solvent DCM-d_2_. In contrast to the corresponding sodium complex, no coalescence of the two CH_2_ signals could be observed up to −90 °C (see Supplementary Figure S2), indicating a more dynamic conformational situation in the lithium cage, which might be related to positional dynamics of the small lithium ion within the cage.

### 2.3. Thermodynamic and Kinetic Properties of **[K^+^•o-Me_2_-1.1.1]BArF^−^**

**Figure 6:**
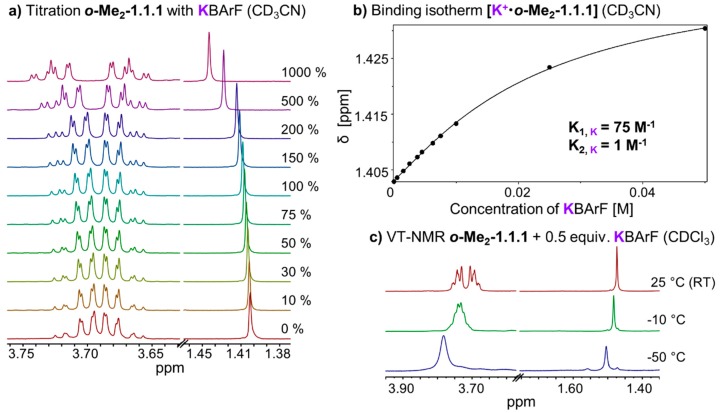
(**a**) Partial ^1^H NMR (400 MHz, 298 K, CD_3_CN) stack plot of ***o*-Me_2_-1.1.1** (5 mM) titration with KBArF from 0 to 1000 mol %; (**b**) Fit of ***o*-Me_2_-1.1.1** titration with KBArF and determination of binding constant (K_1_ for 1:1 complex, K_2_ for 1:2 complex, estimated error: <10%); (**c**) Stack plot of partial variable temperature ^1^H NMR (400 MHz, CDCl_3_).

In chloroform solution, complex **[K^+^•*o*-Me_2_-1.1.1]BArF^−^** exhibits fast exchange on the NMR timescale, which stands in stark contrast to the slow exchange found in the corresponding Li- and Na-complexes (Figure 2b–d). We performed titrations of KBArF to the ***o*-Me_2_-1.1.1** cryptand in solvents CDCl_3_ and CD_3_CN (Figure 6a). The binding isotherm for the titration in acetonitrile (Figure 6b) could be fitted using a 1:1 and 1:2 model using program HypNMR [25]. The resulting binding constants (K_A, **K**_) in CD_3_CN are 75 M^−1^ for the 1:1 complex (K_1_) and only approximately 1 M^−1^ for the formation of the 1:2 complex (K_2_). The binding isotherm for the titration in CDCl_3_ could not be fitted using 1:1, 1:2 or 1:3 models. This result suggests that, in chloroform solution, a variety of species may be present which, together with the low binding constant, points towards an *exo* type of metal binding (so far, we were unable to obtain high-quality single crystals of this complex).

We also recorded variable temperature NMR spectra for complex **[K^+^•*o*-Me_2_-1.1.1]BArF**^−^ in solvent chloroform (Figure 6c). Upon cooling below room temperature, the singlet corresponding to the methyl group broadens and the CH_2_ signals start to broaden and overlap. At −50 °C (223 K), which is the lower limit for solvent chloroform, we observed one relatively broad peak for the CH_2_ groups indicating that the coalescence point has not (yet) been reached at this temperature [33]. For the VT NMR full plot, including more than three temperatures, please refer to Supplementary Figure S3.

### 2.4. Effect of Different Counter-Anions

The data presented above was obtained using commercially-available metal salts of known purity, which we could trust to be acid-free (important for the stability of cryptand). Nevertheless, we wanted to investigate whether different counter-anions have an effect on the strength of metal binding, which is why we conducted three further ^1^H NMR titrations with metal salts NaBF_4_, LiBF_4_ and LiBArF (prepared from AgBArF). The corresponding ^1^H NMR titration plots, as well as the fits of the binding isotherms are shown in the Supplementary Information (Figures S6–S12). The binding constants are summarized in Table 1 and it is clear from this data that the nature of the counter-anion only has a limited effect on the strength of metal binding. Importantly, when comparing salts with the same counter-anion (e.g., BF_4_^−^ or BArF^−^), the relative magnitude of the binding constants remains the same, *i.e.*, lithium binds slightly stronger than sodium, irrespective of the counter-anion (see also discussion in Section 2.5).

A second factor that could affect the binding constants is the presence of water, which is why solvent acetonitrile was kept anhydrous by storing over MS 3 Å (^1^H NMR confirms negligible water content). However, it is virtually impossible to completely dry some of the metal salts used in this study (especially the case for lithium salts), so in many experiments moisture is introduced during the titration. Nevertheless, we are convinced that these low levels of moisture have no strong effect on the binding constants, because the binding isotherms can be fitted very well and because the experimental data is highly consistent for different salts (despite, e.g., LiTPFPB and LiBF_4_ differing significantly in hygroscopy).

### 2.5. Discussion of the Results

The main purpose of this study was to compare the three complexes [**Na^+^**⊂***o*-Me_2_-1.1.1]BArF^−^**, **[Li^+^**⊂***o*-Me_2_-1.1.1]TPFPB^−^** and **[K^+^•*o*-Me_2_-1.1.1]BArF^−^** regarding the thermodynamics and kinetics of metal binding. Table 1 gives an overview on our key findings.

**Table 1:** Summary of key results. Binding constants K_1_ (1:1 complex) and K_2_ (1:2 complex, two hosts/one ion) determined by ^1^H NMR titration experiments in MeCN-d_3_ (estimated error: 10%–20%). Exchange rate k_obs_ determined by EXSY NMR spectroscopy in CDCl_3_ (error *ca.* 2%).

	K_1_ (MeCN-d_3_)	K_2_ (MeCN-d_3_)	k_obs_ (CDCl_3_)
**NaBArF**	1330 M^−1^	10 M^−1^	1.23 s^−1^
**NaBF_4_**	950 M^−1^	10 M^−1^	-
**LiTPFPB**	2750 M^−1^	10 M^−1^	1.99 s^−1^
**LiBArF**	1650 M^−1^	10 M^−1^	-
**LiBF_4_**	2290 M^−1^	10 M^−1^	-
**KBArF**	75 M^−1^	1 M^−1^	high

Regarding the thermodynamics of alkali metal binding, we had previously observed that in solvent chloroform, binding of sodium ions is strong and the addition of one equivalent of NaBArF to **[Li^+^**⊂***o*-Me_2_-1.1.1]TPFPB^−^** results in NMR shifts indicative for near-quantitative replacement of the lithium guest by sodium. In this study, we could now show that, in solvent acetonitrile, excellent fitting of the binding isotherms for all three studied metals is possible, as long as a 1:1 and 1:2 model is taken into account. Of note, the 1:1 complex is dominating in all three cases and the small contribution of the 1:2 model merely improves the fit of the “early” part of the isotherm. When comparing the three studied metals, the binding constants for lithium and sodium are on the same order of magnitude, whereas potassium binding was found to be significantly weaker (see Table 1). This data suggests that sodium (evidence: X-ray structure) and lithium (evidence: ^1^H/^7^Li-Hetero-NOE NMR spectroscopy) bind the cage in an *endo* fashion, whereas potassium binds in an *exo* fashion, resulting in a lower binding constant and, presumably, a highly dynamic binding situation in solution. These results can be convincingly explained by the differences in effective ionic radii of the three studied metals [34]. Simply put, potassium is too large to fit into the cavity of ***o*-Me_2_-1.1.1**, which, when compared to classic cryptands, has a rather rigid structure (no inversion possible at the carbon-bridgeheads).

When drawing a comparison between lithium and sodium, it is interesting to note that ***o*-Me_2_-1.1.1** binds sodium more strongly in chloroform, but lithium more strongly in acetonitrile (although the two binding constants in MeCN-d_3_ are on the same order of magnitude). We can only speculate on the origin of this apparent inversion of metal selectivity. Presumably, sodium is a perfect fit in size for this cryptand, an effect that could dominate in weakly-coordinating solvent chloroform. In acetonitrile, the solvent competes more effectively with the cage and it seems plausible that this competition could give rise to the observed inversion of relative binding strength (according to the HSAB principle “hard” lithium could prefer the “hard” oxygen-donor environment in ***o*-Me_2_-1.1.1** over acetonitrile, while the same effect could be less pronounced for “softer” sodium). A more quantitative explanation could be provided by the solvation energies of the two metal ions in acetontrile, however, published data does not help to explain our observations [35,36], which is why an adequate explanation of this finding will require further studies (e.g., by Isothermal Titration Calorimetry).

Regarding the kinetics of cation binding, our 1D EXSY NMR measurements in CDCl_3_ showed that the degenerate “hopping” of lithium and sodium ions from one cryptand to another is relatively slow (around 1 Hz). The fact that the rate for lithium is twice as fast as that for sodium is in line with the stronger thermodynamic binding of sodium to the cage in solvent chloroform (the stronger binding cation also exchanges more slowly). For potassium, exchange is several orders of magnitude faster (fast on the NMR timescale in both chloroform and acetonitrile), which strongly suggests *exo* binding of potassium to the cage. In our previous communication [22], we reported the interesting observation that addition of crown ether 15-crown-5 led to fast exchange even for metals lithium and sodium, while the same effect did not occur when a classic cryptand was added. We believe that this finding indicates that a crown ether (but not a Lehn-type or orthoester cryptand) can accelerate cation “hopping”, presumably through an associative mechanism [37].

When comparing the results of our VT-NMR experiments, the most interesting finding is that we do observe coalescence for at least one of the CH_2_ signals in the sodium complex, but not in the lithium complex, even when cooling down to the limit of the solvent (DCM-d_2_). We believe that this behavior could be due to an ideal size fit (supported by preliminary DFT calculations) of sodium, which effectively “rigidifies” the organic framework. As we have shown, lithium does also bind strongly to the cage, but due to the smaller size of the lithium cation, the binding situation is likely much more dynamic. These dynamics could result in a less effective rigidification of the cage which, in turn, could explain the lack of an observed coalescence of the CH_2_ signals at low temperature. For the case of the potassium complex, we studied a 1:1 mixture of **[K^+^•*o*-Me_2_-1.1.1]BArF^−^** and ***o*-Me_2_-1.1.1** by VT NMR with the goal of determining a rate constant of cation exchange for this complex. However, in solvent chloroform (necessary to draw direct comparisons) no coalescence was observed down to the limit −55 °C.

## 3. Experimental Section

### 3.1. Reagents and Instruments

All commercially-purchased reagents were used without further purification. Molecular sieves were dried by heating for 3 days at 150 °C under reduced pressure (10^−2^ mbar). All solvents were dried over molecular sieves for at least 24 h. All orthoester exchange reactions (catalyzed by TFA) were carried out under nitrogen. After the acid is quenched (e.g., by addition of triethylamine), many of the orthoesters described herein were found to be unusually stable against water. NMR solvents CDCl_3_, and CD_3_CN were stored over molecular sieves. NMR spectra were recorded on Bruker Avance 400 (^1^H: 400 MHz) and Jeol Alpha 500 (^1^H: 500 MHz) spectrometers at 298 K and referenced to the residual solvent peak (^1^H: CDCl_3_, 7.24 ppm; CD_3_CN, 1.94 ppm).

### 3.2. Experimental Procedures

#### 3.2.1. Synthesis of Starting Materials

##### Preparation of Stock Solutions

To achieve a high level of stoichiometric accuracy and adequate exclusion of moisture, metal salts and diethylene glycol were added from stock solutions that were dried over molecular sieves prior to addition of orthoester and acid catalyst.

For the salt stock solution, sodium tetrakis[3,5-bis(trifluoromethyl)phenyl]borate (NaBArF, 0.14 mmol, 127.8 mg) and diethylene glycol (DEG, 0.42 mmol, 39.9 µL) were dissolved in CDCl_3_ (14 mL) and dried over 4 Å molecular sieves (*ca.* 1 g) for 3 days.

To obtain the acid stock solution, trifluoroacetic acid (TFA, 240 µmol, 18.4 µL) was topped up with CDCl_3_ to obtain a total volume of 2 mL.

##### Synthesis of **[Na^+^**⊂***o*-Me_2_-1.1.1]BArF^−^**

Molecular sieves 4 Å (1 g) were added to 6.0 mL of the salt stock solution and the reaction mixture was left to stand at room temperature for 16 h. The orthoester (0.12 mmol) and 10 µL of the acid stock solution (1 mol %) was added and the reaction mixture was shaken. Every 24 h, 10 µL of the acid stock solution (1 mol %) was added to keep the exchange reaction active (molecular sieves slowly transform the acid catalyst into inactive anhydride and/or esters) and the reaction progress was monitored regularly by ^1^H NMR spectroscopy. [**Na^+^**⊂***o*-Me_2_-1.1.1]BArF^−^** was prepared from NaBArF, trimethyl orthoacetate and diethylene glycol. After 5 days, the solvent was removed under reduced pressure and the title compound was obtained as a colorless solid in 67% yield. Further purification could be achieved by passing a solution of the crude mixture in chloroform through a short plug of silica gel or by crystallization (e.g., slow vapor diffusion of cyclopentane). Comprehensive characterization data was published previously [22].

##### Synthesis of ***o*-Me_2_-1.1.1**

**[Na^+^**⊂***o*-Me_2_-1.1.1]BArF^−^** (~2 mg) was stirred in CDCl_3_ with chloride exchange resin (Lewatite^®^ MP-64, 300 mg) for 6 h at RT. The reaction progress was monitored by ^1^H NMR spectroscopy and the anion exchange resin was removed by filtration through a syringe filter once the reaction was complete. After removal of the solvent under reduced pressure, the title compound was obtained quantitatively as a colorless oil. Characterization data was published previously [22].

##### Synthesis of **[Li^+^**⊂***o*-Me_2_-1.1.1]TPFPB^−^** and **[K^+^•*o*-Me_2_-1.1.1]BArF^−^**

Cryptand ***o*-Me_2_-1.1.1** was prepared as described above (in solvent chloroform). A quantitative amount of lithium tetrakis(pentafluorophenyl) borate ethyl etherate (LiTPFPB) or potassium tetrakis[3,5-bis(trifluoromethyl)phenyl]borate (KBArF) was added from a stock solution (KBArF from an acetonitrile stock solution) or as solid compound to obtain the desired cryptate. The complete solvent was removed under vacuum to dry the sample. The sample was keep under nitrogen and dry deuterated solvent was added to obtain the desired sample for the NMR experiments. Characterization data was published previously [22].

#### 3.2.2. General Procedure for ^1^H NMR Titrations

The cryptand ***o*-Me_2_-1.1.1** was always freshly prepared as described above. The precise quantity of ***o*-Me_2_-1.1.1** in CDCl_3_ solution was determined with 1,4-dinitrobenzene as internal standard. For titration experiments a 5 mM ***o*-Me_2_-1.1.1** solution was prepared via dilution. CDCl_3_ was removed under reduced pressure and subsequently replaced by CD_3_CN, for titrations in CD_3_CN. The concentrations of the titrated salt solutions (NaBF_4_, NaBArF, LiBF_4_, LiBArF, LiTPFPB and KBArF) were 75 mM with 5 mM ***o*-Me_2_-1.1.1** added to keep the concentration of the cryptand constant throughout the titration.

#### 3.2.3. General Procedure for ^1^H-1D EXSY NMR Experiments

The cryptand ***o*-Me_2_-1.1.1** was always freshly prepared as described above. The amount of ***o*-Me_2_-1.1.1** in CDCl_3_ solution was determined with 1,4-dinitrobenzene as internal standard. For 1D EXSY NMR experiments a 5 mM ***o*-Me_2_-1.1.1** solution (600 µL) was prepared and 1.5 μmol of the corresponding salt was added to this solution to obtain a 1:1 mixture of cryptate and cryptand. The spectra were measured at room temperature with different mixing times from 10 ms up to 1200 ms. The quotient of the integrals from the EXSY peak (I_(X)_) and excited peak (I_(d)_) are plotted *versus* the mixing time (Figure 3d and Figure 5d). These points were fitted according to the following function (r: exchange rate) [28,29,30].

(1) $$\frac{I\left(x\right)}{I\left(d\right)}=\frac{1-e^{\left(-r\;\times \;x\right)}}{1+e^{\left(-r\;\times \;x\right)}}\;$$

(2) $$r=k_{1}+\;k_{-1}$$

(3) $$k_{obs}=\;k_{1}\;=\;k_{-1}=\;\frac{r}{2}$$

#### 3.2.4. Hetero-NOE NMR Spectroscopy

For ^1^H/^7^Li HOESY NMR experiments [32], a 5 mM ***o*-Me_2_-1.1.1** solution (600 µL) was prepared and 3.0 μmol of the LiTPFPB salt was added to this solution to obtain a 5 mM solution of **[Li^+^**⊂***o*-Me_2_-1.1.1]TPFPB^−^** in CDCl_3_. The spectrum was recorded at room temperature with a mixing time of 800 ms after degassing the sample.

## 4. Conclusions

In this study, we disclose thermodynamic and kinetic data on three novel orthoester complexes **[Na^+^**⊂***o*-Me_2_-1.1.1]BArF^−^**, **[Li^+^**⊂***o*-Me_2_-1.1.1]TPFPB^−^** and **[K^+^•*o*-Me_2_-1.1.1]BArF^−^**. Our key findings include the observation that, in solvent acetonitrile, the binding constants for sodium and lithium are moderate (compared to classic Lehn-type cryptands) and that the binding constant for potassium is approximately two orders of magnitude lower, which is very likely a result of an *exo* mode of binding. A pronounced “cutoff” between *endo*-binding lithium and sodium on the one hand and *exo*-binding potassium on the other hand was also found to be present in the kinetics of degenerate cation exchange in chloroform, which are slow for the two former metals and fast for the latter.

These results have two important implications concerning future directions of research on orthoester cryptands: (i) for applications in the area of drug delivery, cages with higher binding constants in polar solvents such as water or DMSO are needed. Due to their thermodynamically-controlled one-step synthesis, such superior hosts could be easily accessible by using different diol “ligands” for the self-assembly reaction; and (ii) The observation of a sharp selectivity for small metals lithium and sodium over larger potassium (higher selectivity than in classic Lehn-type cryptands) [7] indicates that these cages could be ideally suited for performing subcomponent self-sorting experiments, in which metal ions would effectively select their preferred host compounds from mixtures of building blocks.

## Supplementary Material

Supplementary materials can be found at http://www.mdpi.com/1422-0067/16/09/20641/s1.
