# Supplementary material for: NMR Studies on Li+, Na+ and K+ Complexes of Orthoester Cryptand o-Me2-1.1.1
Source: Int J Mol Sci. 2015 Aug 31;16(9):20641–56. doi: 10.3390/ijms160920641 (PMC4613223; doi:10.3390/ijms160920641)
Supplement: Supplementary file 1 [file ijms-16-20641-s001.pdf]

Supplementary Information

Variable Temperature NMR (VT NMR) Spectra

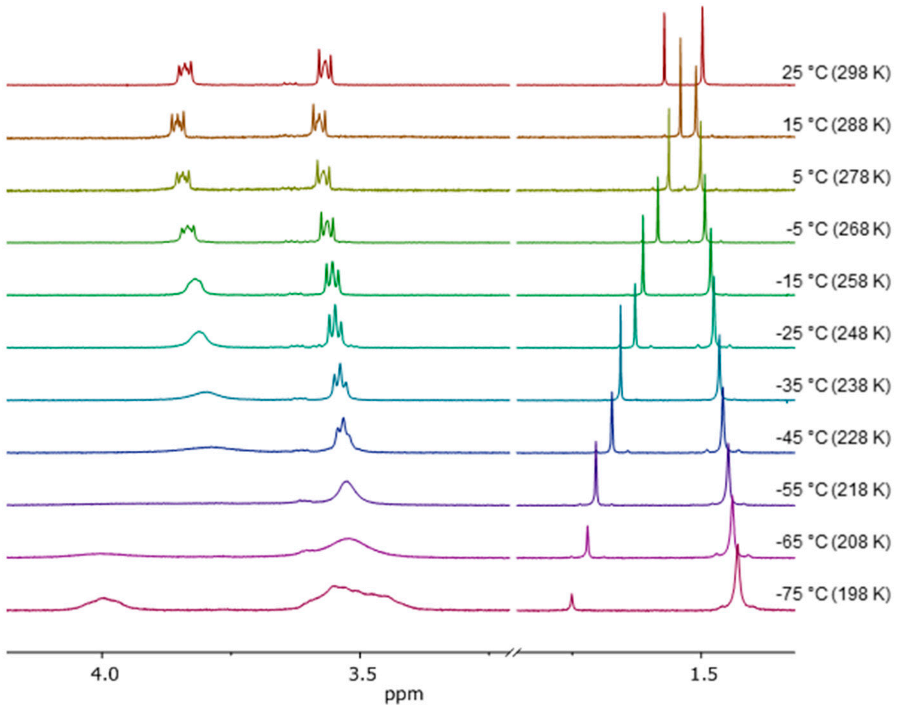

Figure S1. Partial ^{1}H NMR spectra (400 MHz, CD_2Cl_2) of **[Na^{+}⊂*o*-Me$_2$-1.1.1]BArF^{-}**.

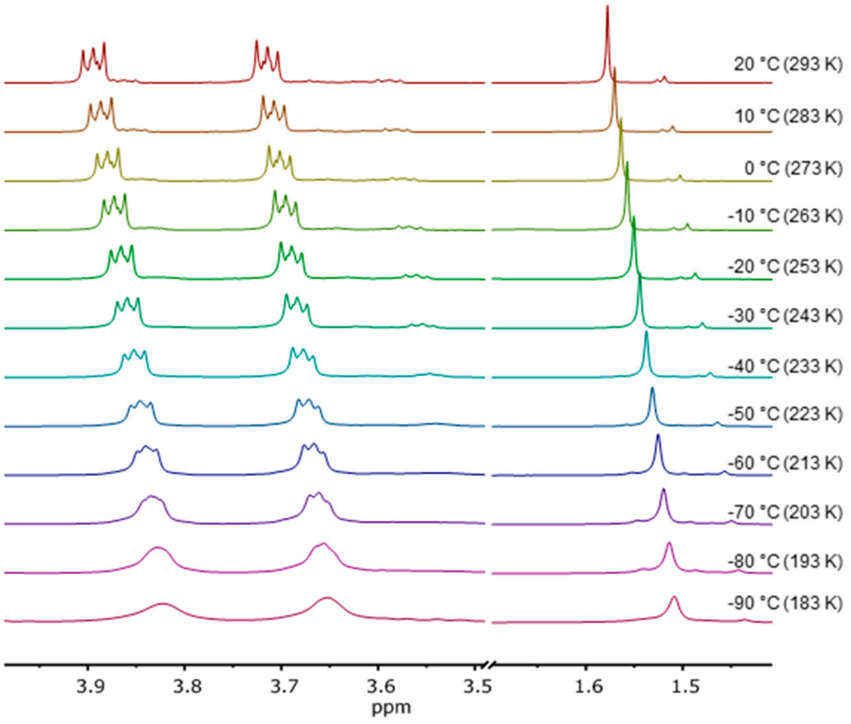

Figure S2. Partial ^{1}H NMR spectra (400 MHz, CD_2Cl_2) of **[Li^{+}⊂*o*-Me$_2$-1.1.1]TPFPB^{-}**.

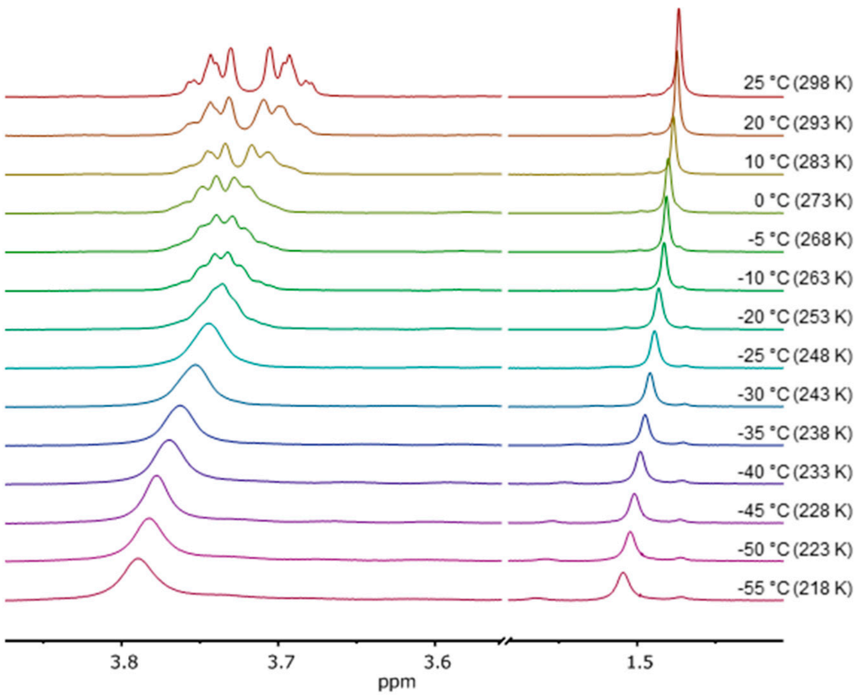

Figure S3. Partial ^{1}H NMR spectra (400 MHz, $CDCl_3$) of **[K^{+}•*o*-Me_2-1.1.1]BArF^{-}**.

Titration Plots

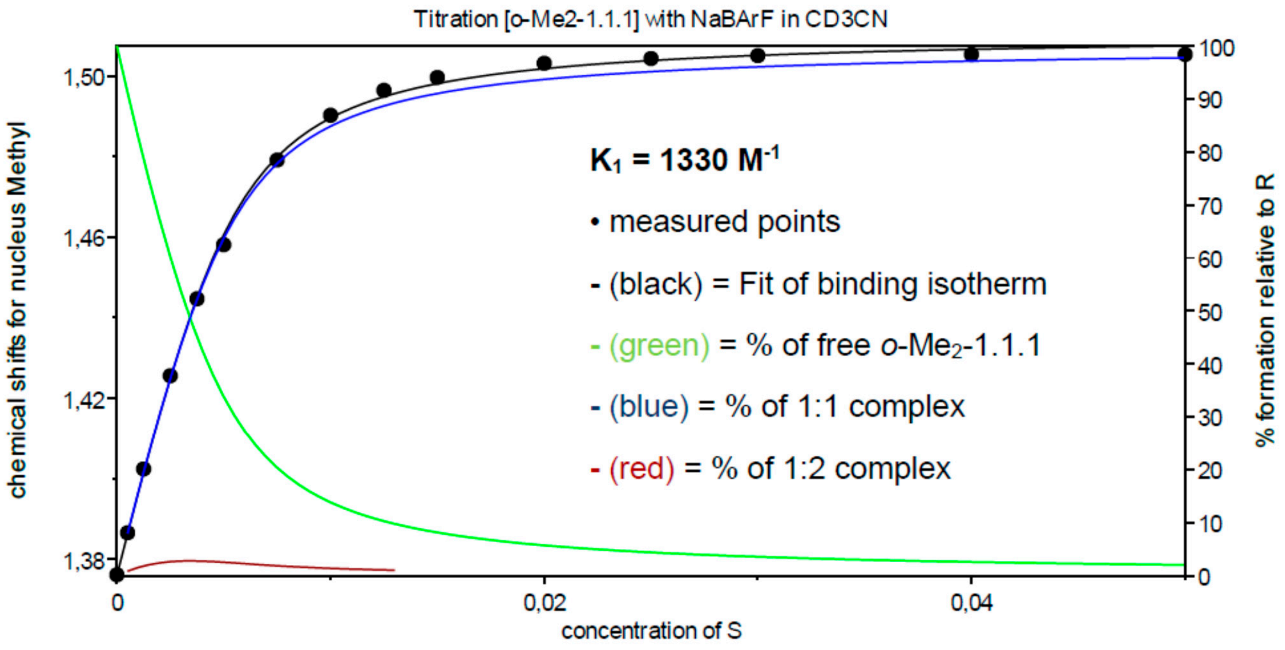

Figure S4. Binding isotherm with plot of different species for titration of ***o*-Me_2-1.1.1** (5 mM) with NaBArF from 0 to 1000 mol % in CD_3CN (below 1% HypNMR does not plot species).

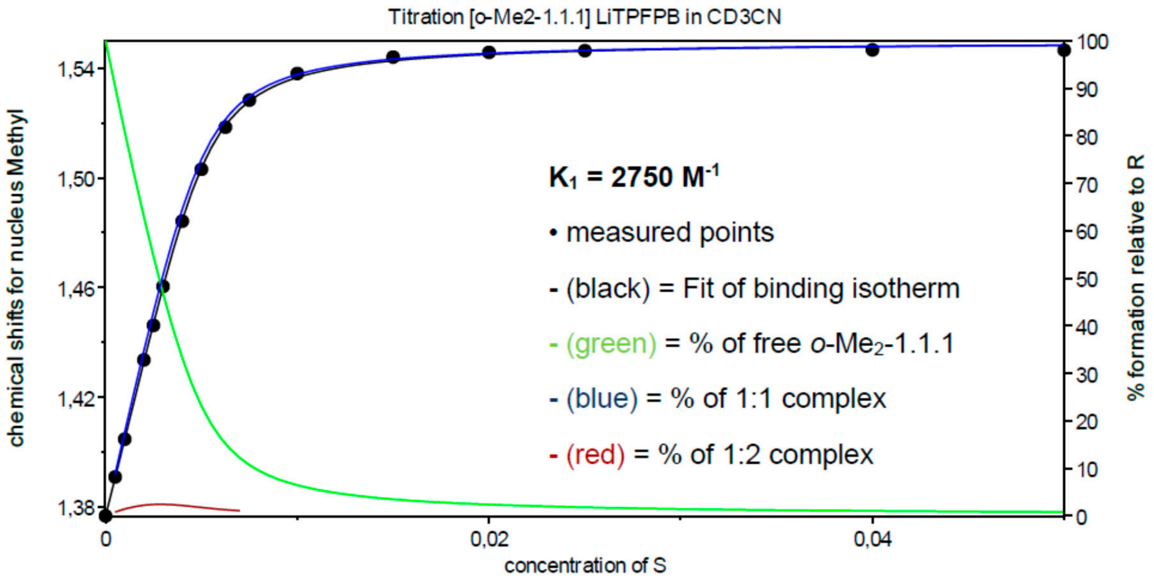

Figure S5. Binding isotherm with plot of different species for titration of ***o*-Me$_2$-1.1.1** (5 mM) with LiTPFPB from 0 to 1000 mol % in CD_3CN (below 1% HypNMR does not plot species).

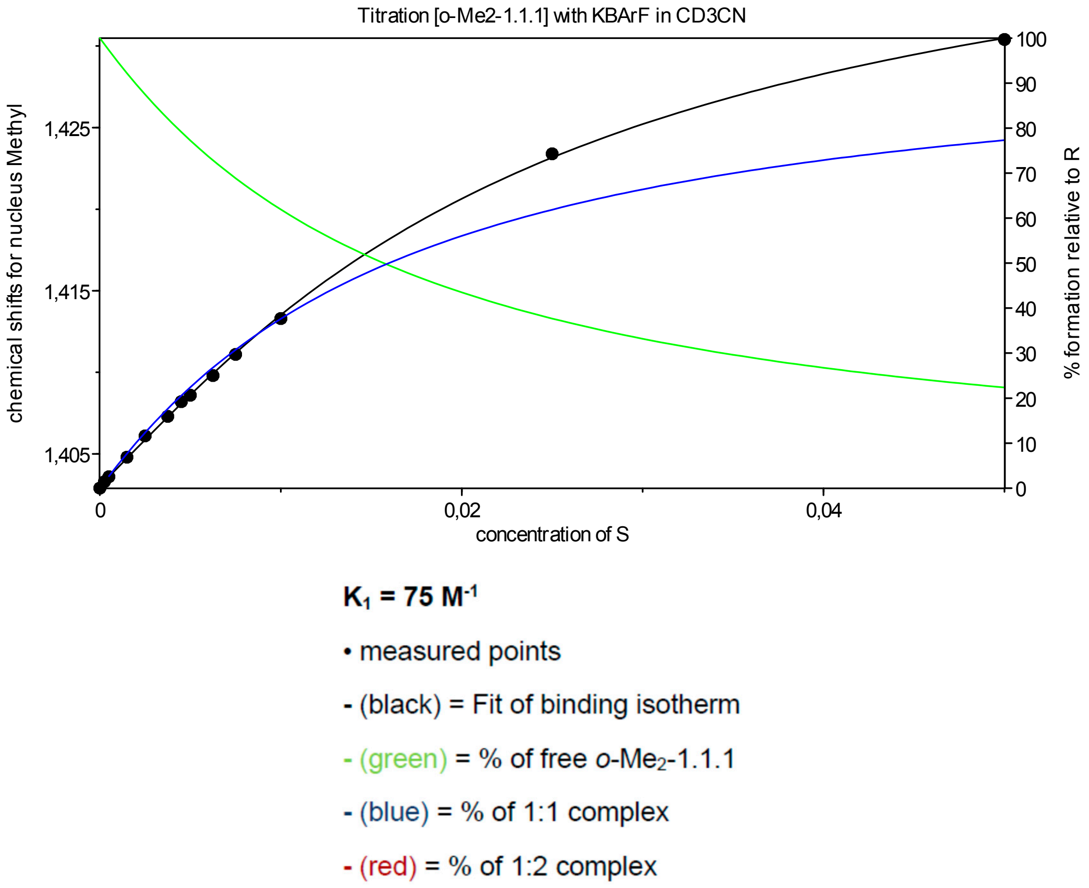

Figure S6. Binding isotherm with plot of different species for titration of ***o*-Me$_2$-1.1.1** (5 mM) with KBArF from 0 to 1000 mol % in CD_3CN (below 1% HypNMR does not plot species, which is why there is no red line shown).

Titration of *o*-Me$_2$-1.1.1 (5mM) with NaBF$_4$

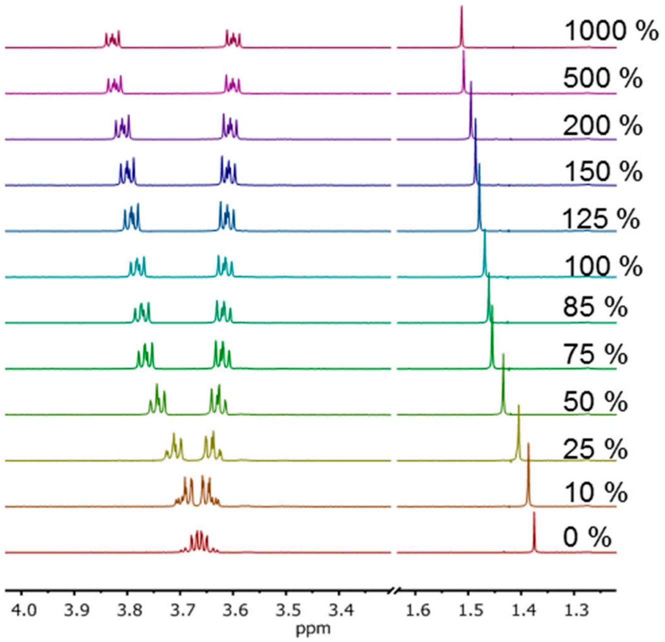

Figure S7. Partial ^{1}H NMR (400 MHz, 298 K, CD_3CN) stack plot of ***o*-Me$_2$-1.1.1** (5 mM) titration with $NaBF_4$ from 0 to1000 mol %.

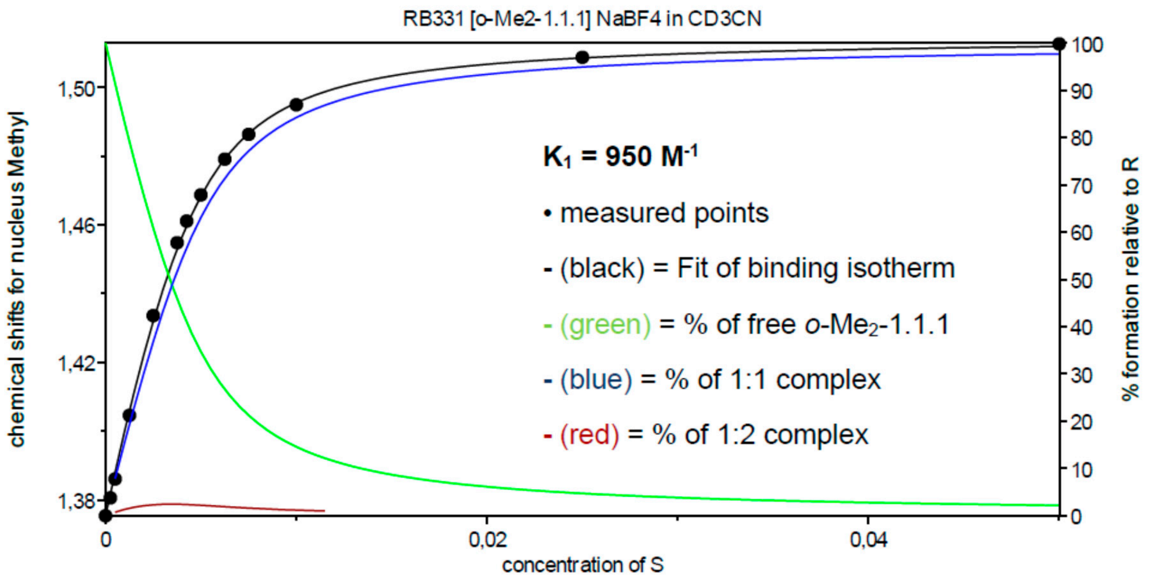

Figure S8. Binding isotherm with plot of different species for titration of ***o*-Me$_2$-1.1.1** (5 mM) with $NaBF_4$ from 0 to 1000 mol % in CD_3CN (below 1% HypNMR does not plot species).

Titration of *o*-Me_2-1.1.1 (5mM) with $LiBF_4$

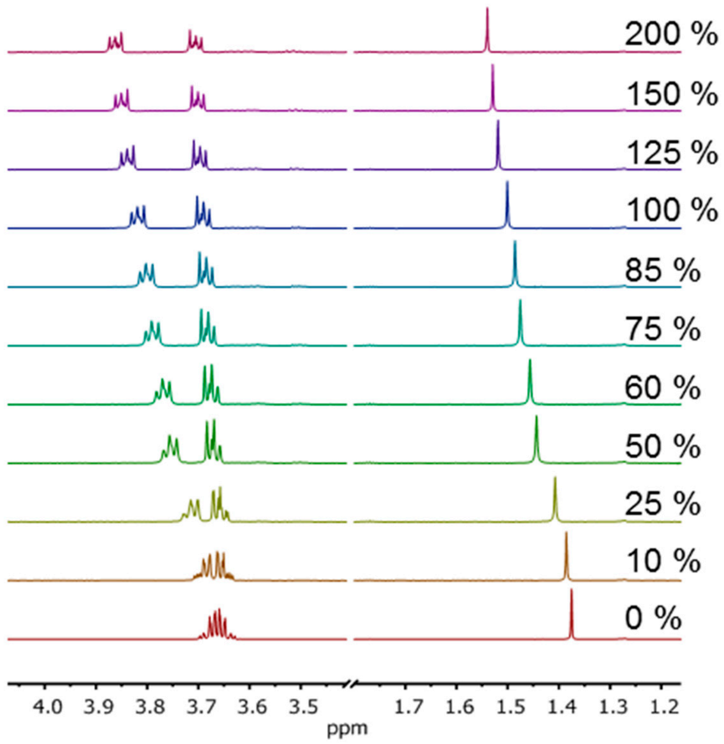

Figure S9. Partial ^{1}H NMR (400 MHz, 298 K, CD_3CN) stack plot of ***o*-Me_2-1.1.1** (5 mM) titration with $LiBF_4$ from 0 to 200 mol %. (After addition of 200 mol % salt, noticeable hydrolysis of the cage occurred (most likely $LiBF_4$ is the most acidic of the salts used), which is why the fit of the binding isotherm was based only on these "early" titration points.)

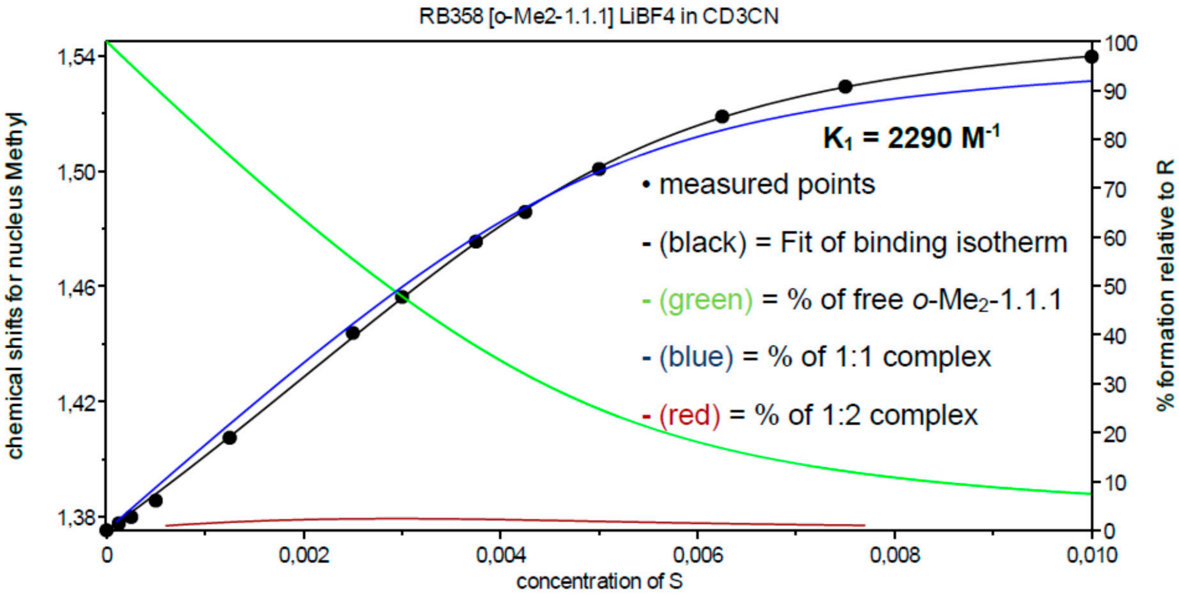

Figure S10. Binding isotherm with plot of different species for titration of ***o*-Me_2-1.1.1** (5 mM) with **$LiBF_4$** from 0 to 500 mol % in CD_3CN (below 1% HypNMR does not plot species).

Titration of *o*-Me$_2$-1.1.1 (5mM) with LiBArF

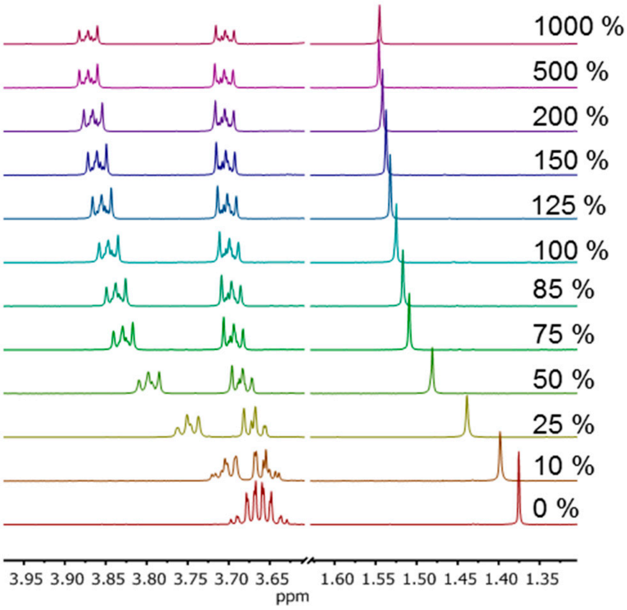

Figure S11. Partial ^{1}H NMR (400 MHz, 298 K, CD_3CN) stack plot of ***o*-Me$_2$-1.1.1** (5 mM) titration with LiBArF from 0 to 1000 mol %.

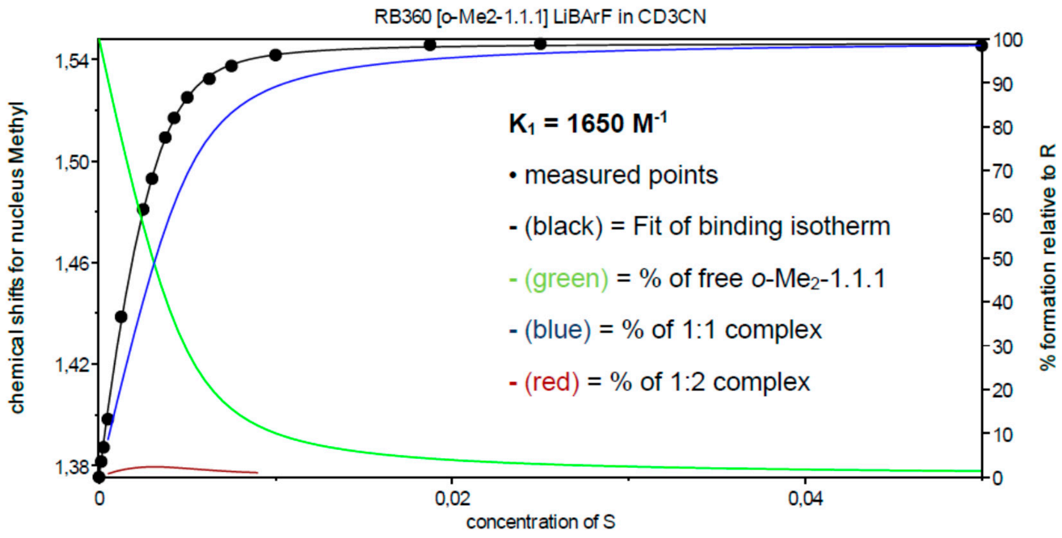

Figure S12. Binding isotherm with plot of different species for titration of ***o*-Me$_2$-1.1.1** (5 mM) with LiBArF from 0 to 1000 mol % in CD_3CN (below 1% HypNMR does not plot species).
